# Systemic and functional effects of continuous azithromycin treatment in patients with severe chronic obstructive pulmonary disease and frequent exacerbations

**DOI:** 10.3389/fmed.2023.1229463

**Published:** 2023-07-24

**Authors:** Ester Cuevas, Daniel Huertas, Concepción Montón, Alicia Marin, Anna Carrera-Salinas, Xavier Pomares, Marian García-Nuñez, Sara Martí, Salud Santos

**Affiliations:** ^1^Department of Respiratory Medicine, Hospital Universitari de Bellvitge, Institut d’Investigacio Biomedica de Bellvitge – IDIBELL, Universitat de Barcelona, L’Hospitalet de Llobregat, Barcelona, Spain; ^2^Department of Respiratory Medicine, Consorci Sanitari Alt Penedès Garraf, Barcelona, Spain; ^3^Department of Respiratory Medicine, Hospital de Sabadell, Institut Universitari Parc Taulí-UAB, Sabadell, Spain; ^4^Research Network for Respiratory Diseases (CIBERes), ISCIII, Madrid, Spain; ^5^Department of Respiratory Medicine, Hospital Germans Trias i Pujol, Institut d’Investigació en Ciències de la Salut Germans Trias i Pujol – IGTP, Universitat Autònoma de Barcelona, Badalona, Spain; ^6^Department of Microbiology, Hospital Universitari de Bellvitge, IDIBELL, Universitat de Barcelona, Barcelona, Spain

**Keywords:** inflammatory markers, interleukins, exacerbations, COPD, azithromycin, microorganisms

## Abstract

**Background:**

Continuous treatment with azithromycin may lead to fewer acute exacerbations of chronic obstructive pulmonary disease (AECOPD), but little is known of its impact on systemic and functional outcomes in real-life settings.

**Methods:**

This was a multicenter prospective observational study of patients with severe COPD who started treatment with azithromycin. Tests were compared at baseline and after 3 and 12 months of treatment. These included lung function tests, a 6-min walking test (6MWT), and enzyme-linked immunosorbent assays of serum and sputum markers, such as interleukins (IL-6, IL-8, IL-13, IL-5), tumor necrosis factor receptor 2 (TNFR2), and inflammatory markers. Incidence rate ratios (IRR) and their 95% confidence intervals (95% CI) are reported.

**Results:**

Of the 478 eligible patients, the 42 who started azithromycin experienced reductions in AECOPDs (IRR, 0.34; 95% CI, 0.26–0.45) and hospitalizations (IRR, 0.39; 95% CI, 0.28–0.49). Treatment was also associated with significant improvement in the partial arterial pressure of oxygen (9.2 mmHg, 95% CI 1.4–16.9) at 12 months. While TNFR2 was reduced significantly in both serum and sputum samples, IL-13 and IL-6 were only significantly reduced in serum samples. Moreover, an elevated serum and sputum IL-8 level significantly predicted good clinical response to treatment.

**Conclusion:**

Continuous azithromycin treatment in a cohort of patients with severe COPD and frequent exacerbations can significantly reduce the number and severity of exacerbations and improve gas exchange. Treatment changes the pattern of microorganism isolates and decreases the inflammatory response. Of note, IL-8 may have utility as a predictor of clinical response to azithromycin treatment.

## 1. Introduction

Chronic obstructive pulmonary disease (COPD) is an inflammatory and systemic disease that affects the airways. Acute exacerbations of COPD (AECOPD) affect not only the natural history of the disease but also quality of life, the number of hospitalizations, and mortality rates (1). Exacerbations, which are frequently caused by respiratory viral or bacterial infections, are events characterized by increases in respiratory symptoms that worsen in <14 days, may be accompanied by tachypnea and/or tachycardia, and are often associated with increased local and systemic inflammation (2). Various interventions have been shown to prevent AECOPDs, including both non-pharmacological measures (e.g., quitting smoking, respiratory rehabilitation, and vaccines) and pharmacological measures (e.g., bronchodilator and corticosteroid inhalers) (3). Nevertheless, 25–30% of patients still experience AECOPD, indicating that these measures do not prevent all cases (4).

Randomized controlled trials have demonstrated the efficacy of prophylactic azithromycin in reducing AECOPDs, and as such, current clinical guidelines recommend its use in patients with frequent exacerbations despite optimal inhaled therapy (5–7). However, there is limited evidence on the most appropriate time of administration or the most effective dose needed to control exacerbations and minimize side effects. Albert et al. compared the use of 250 mg of azithromycin per day for 1 year irrespective of COPD severity or frequency of AECOPD. They showed both an increased time lag between exacerbations and a decrease in the frequency of exacerbations compared to placebo (5). The COLUMBUS trial also showed that 500 mg of azithromycin three times a week for 1 year significantly reduced (42%) the exacerbation rate compared with placebo in patients with three or more AECOPDs annually (6). Finally, a more recent study found that doses of 250 mg and 500 mg three times a week had comparable effectiveness at preventing exacerbations in patients with severe COPD and frequent exacerbations, and that this could minimize the potential side effects of a long-term antibiotic therapy (7).

Antibiotic prophylaxis in AECOPDs may increase the risk of selection for resistant bacteria, facilitating the spread of antimicrobial resistance. Even though azithromycin is usually well-tolerated drug, undesirable side effects can still result from polypharmacy, advanced age, and comorbidities in real-world settings. Moreover, the therapeutic response can vary depending on the characteristics and baseline statuses of patients. Investigating what factors predict a favorable response, both in the number of exacerbations and their functional effects, is of particular clinical interest.

This study aimed to analyze the systemic and pulmonary functional changes associated with severe COPD in patients with frequent exacerbations after continuous azithromycin treatment, their effect on microbiological isolates in residual exacerbations, and the potential involvement of inflammatory and immunomodulatory mechanisms.

## 2. Methods

### 2.1. Study design

This was a multicenter, observational, and prospective study of patients with severe COPD and frequent exacerbations who regularly attended comprehensive care programs in the respiratory day hospitals of three university hospitals in Barcelona. The participating hospitals were Hospital Universitari de Bellvitge (HUB), Hospital Parc Taulí (HPT), and Hospital Germans Trias i Pujol (HGTiP). All patients who started treatment with azithromycin according to clinical guideline recommendations and practice (8) were included consecutively between January and December, 2017.

### 2.2. Patient selection

COPD was defined according to the GOLD recommendations: a history of smoking (>10 pack-years) and an FEV1 (forced exhalation volume in 1 second) to FVC (forced vital capacity) ratio < 70% (8). By clinical consensus, and with the objective of homogenizing the selection between the different centers, we included patients at the maximum therapeutic step in the 6 months prior to inclusion if they had frequent exacerbations, defined as ≥4 moderate AECOPDs in the last year (≥3 if two were severe), and had sputum cultures negative for mycobacteria. Patients with contraindications to macrolide therapy (allergy, long QT syndrome), different respiratory pathologies, lack of suitability for macrolide treatment, according to the clinician’s criteria (severe cardiovascular disease, hearing loss, use of co-therapies that prolong the QT interval or drug interactions) and prior use of azithromycin or inhaled colimycin (not concomitant initiation) were excluded.

All patients signed an informed consent form in accordance with the principles outlined in the Declaration of Helsinki, and the local ethics committee approved the study (CEIC, ref. SSP-AZI-2016-01).

### 2.3. Visits and follow-up

All included patients started treatment with azithromycin 250 mg three times per week for 1 year. Sociodemographic data, anthropometrics, COPD history, and symptom evaluations were collected in all patients. Symptom tests included the COPD Assessment Test (CAT), London Chest Activity of Daily Living Scale (LCADL) (9), and the modified Medical Research Council (mMRC) Dyspnea Scale. In addition, participants underwent a 6-min walking test (6MWT), respiratory functional test, and arterial blood gas analyzes at baseline and after 3 and 12 months of follow-up. Blood and sputum samples were collected at all visits for laboratory determinations. All moderate and severe exacerbations were recorded and treated according to appropriate criteria and guidelines. Exacerbations from the year before starting treatment were also registered. An exacerbation was defined as any event that caused a worsening of respiratory symptoms and that required modification of the patient’s usual treatment.

### 2.4. Quantification of inflammatory parameters in blood and sputum

Serum samples were obtained from venous blood. After allowing the sample to stand at room temperature for 2 h, it was centrifuged for 15 min at 1000 × *g*, and the supernatant was taken for analysis.

Sputum was separated from contaminating saliva by macroscopic examination, before being weighed and homogenized with 4 volumes of dithiothritol (Sputasol, Oxoid Ltd., Hants, United Kingdom). After 15 min at room temperature, the same volume of phosphate-buffered saline was added and the whole mixture was filtered and centrifuged at 600 × *g* for 15 min. The supernatant was stored at −80°C for determinations.

Analysis was performed by enzyme-linked immunosorbent assay (ELISA). Cytokine concentrations (IL-5, IL-6, IL-8, IL-13) were measured in serum samples and supernatant sputum samples using a multiplex immune-bead assay (Milliplex MAP High Sensitivity Human T cell panel Kit; Merck Millipore). TNR2 was analyzed with a Human soluble TNF Receptor 2 ELISA Kit (sTNFRII; RAB0490). All procedures were performed according to the manufacturer’s instructions, centralized in one of the centers.

### 2.5. Sputum collection and bacterial load detection

Sputum samples were obtained spontaneously at visits and during AECOPD episodes before starting any antimicrobial treatment. Only good quality sputum samples were considered (i.e., 25 leukocytes per low-power field) following the microbiological routine (10). Briefly, samples were homogenized with dithiothreitol (Sputasol, Oxoid Ltd., Hants, United Kingdom), and plated onto blood agar, chocolate agar, and MacConkey agar before overnight incubation at 37°C in a 5% CO_2_ atmosphere (blood and chocolate agar) or ambient air atmosphere (MacConkey agar). Bacterial colonies were sub-cultured for identification by matrix-assisted laser desorption/ionization time-of-flight mass spectrometry (Bruker Daltonik GmbH, Bremen, Germany).

### 2.6. Statistical analyzes

Data are expressed as means ± standard deviation (SD) or medians [25th–75th percentile] for continuous data and as frequencies (percentage) for categorical data. Multiple comparisons were evaluated by chi-squared (categorical), student *t* (parametric), or Mann–Whitney (nonparametric) tests, applying the Bonferroni method if the Kruskal–Wallis test found significant differences. A negative binomial regression model was used to study the number of exacerbations after treatment, reporting the incidence rate ratio (IRR) and 95% confidence interval (95%CI). Different predictive statistical models were fitted for each analytical or clinical variable of interest, adjusted by age and Charlson index. A value of p of 0.05 was considered statistically significant. IBM SPSS version 22 (IBM Corp., Armonk, NY, United States) was used for all analyzes.

## 3. Results

### 3.1. Study subjects

**Figure 1:**
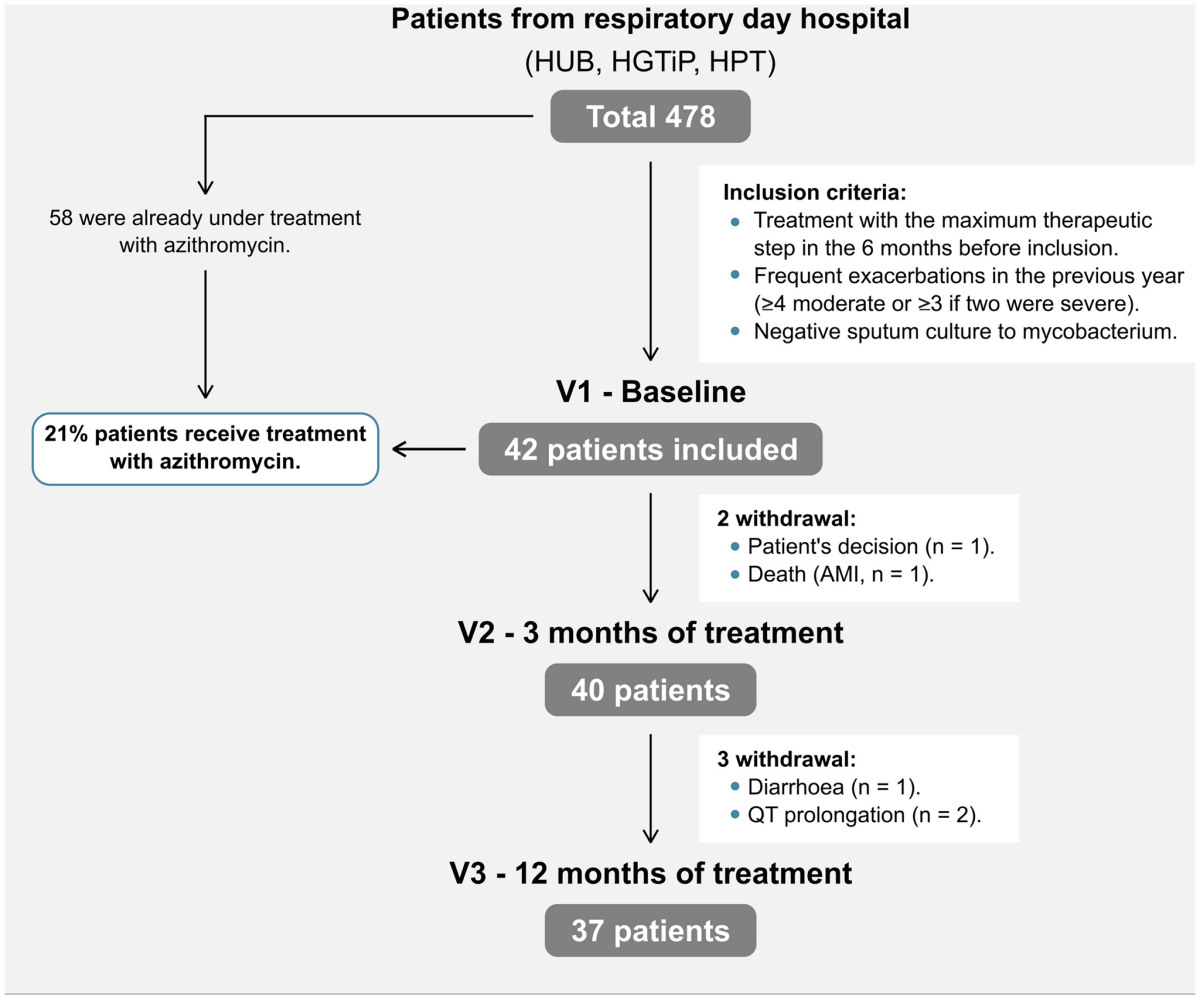
Patient flowchart. AMI, acute myocardial infarction; HUB, Hospital Universitari de Bellvitge; HGTiP, Hospital Germans Trias I Pujol; HPT, Hospital Parc Taulí.

Of the 478 eligible patients with severe COPD in three respiratory day hospitals, we included 42 (8.8%) who started azithromycin (Figure 1; mean age 72.2 ± 7.09 years). Fifty-eight patients were already under treatment with long-term azithromycin therapy and were excluded; therefore, 21% of patients in this cohort were treated. At the time of inclusion, all patients were receiving combined inhaled bronchodilator therapy, long-acting β_2_-agonists (LABAs) and long-acting muscarinic antagonists (LAMAs), 39 (93%) inhaled corticosteroids (ICS) and nine (21%) long-term oxygen therapy. Participants corresponded to group E of the current GOLD classification (8), having severe airflow obstruction (mean FEV_1_ 44.5% ± 13.8%), an average mMRC dyspnea symptom score of 2, and a median of 5 (4–6) exacerbations during the previous year (Table 1). Patient characteristics were comparable among the three participating hospitals. Only 37 patients finished the study, with patients withdrawn due to cardiac pathology (*n* = 3; 1 acute myocardial infarction, 2 QT prolongations), diarrhea (*n* = 1), and personal preference (*n* = 1).

**Table 1:** Clinical and functional characteristics of study patients.

	Total	HUB	HGTiP	HPT	*p*-value
	*n* = 42	*n* = 15	*n* = 13	*n* = 14	
Age, years	72.2 (7.09)	71.5 (4.76)	72.2 (7.91)	73.1 (8.7)	0.838
Male, *n* (%)	38 (90.5)	13 (86.7)	12 (92.3)	13 (92.9)	0.947
BMI, kg/m^2^	27 (5.31)	26.7 (4.48)	28.7 (5.01)	25.7 (6.2)	0.326
Charlson index	2 [1–3]	1 [1–2.75]	2.5 [1.75–4]	1 [1–2]	0.074
Current smokers, *n* (%)	4 (9.52)	0 (0)	3 (23.1)	1 (7.14)	0.174
Pack-years	57.9 (28.9)	59.6 (24.5)	60 (44)	53.9 (17.1)	0.865
FEV_1_, % reference	44.5 (13.8)	40 (7.2)	47.3 (14.3)	49.2 (23.9)	0.407
FEV_1_/FVC, % reference	45.4 (11.5)	39.5 (8.57)	54.7 (8.57)	39 (12)	0.003
RV, % reference	153 (43.5)	165 (46.5)	126 (36.9)	169.4 (30.3)	0.098
DLCO, % reference	45.6 (21)	46.8 (15.5)	54.5 (24.9)	28.8 (18)	0.092
6MWT, meters	341 (107)	326 (106)	371 (124)	333 (97)	0.562
PaO_2_	69.2 (17.07)	72.2 (9.28)	69.3 (28.8)	66.1 (66.1)	0.675
PaCO_2_	42.2 (8.39)	42.4 (6.69)	45.3 (11.3)	40.8 (6.6)	0.378
Dyspnea mMRC	2 [2–2]	2 [2–2]	2 [1–2]	2 [2–2.75]	0.007
CAT	16 [11–21]	16 [11.2–21]	14 [11–19]	17 [11.5–21]	0.749
LCADL	21 [17–29.5]	21 [17–24]	21.5 [18–24]	18 [14–30]	0.579
BQ index	4 [1–6]	2 [0–6.5]	2 [0–7.5]	6 [4.25–10.5]	0.061
Previous exacerbations	5 [4–6]	6 [4–7.5]	4 [4–5]	5 [4.75–5.75]	0.135

Data are presented as mean (SD) or median [25th–75th percentile]. 6MWT, 6-min walking test; BMI, body mass index; BQ index, bronchiectasis index; CAT, COPD Assessment Test; DLCO, diffusing lung capacity for carbon monoxide; Dyspnea mMRC, dyspnea scale modified Medical Research Council; FEV_1_, forced expiratory volume in one second; FVC, forced vital capacity; HUB, Hospital Universitari de Bellvitge; HGTiP, Hospital Germans Trias I Pujol; HPT, Hospital Parc Taulí; LCADL, London chest activity of daily living scale; PaO_2,_ partial arterial pressure of oxygen; PaCO_2,_ partial arterial pressure of carbon dioxide; RV, residual volume; SD, standard deviation; *p < 0.05 significant.

### 3.2. Clinical and functional effects after continuous azithromycin treatment

A significant improvement in the partial arterial pressure of oxygen (PaO_2_) was observed after treatment with azithromycin at 3 and 12 months compared with the baseline visit (9.21 mmHg increase at 12 months; 95%CI, 1.45–16.97). This was also associated with a significant improvement in oxygen saturation both at rest and during exercise. However, symptoms (except for a slight improvement in CAT and LCADL scores), the 6MWT distance, and lung function did not differ significantly after 12 months (Table 2).

**Table 2:** Clinical and functional effects of continuous azithromycin in patients with severe COPD.

Parameters	Baseline	3 months	12 months	*p*-value
Dyspnea mMRC, *n* (%)				0.697
1	7 (18.9)	7 (18.9)	7 (19.4)	
2	26 (70.3)	23 (62.2)	23 (63.9)	
3	4 (10.8)	7 (18.9)	6 (16.67)	
CAT	16.2 (6.2)	14.5 (4.8)	13.9 (4.8)	0.059
LCADL	23.9 (12.7)	23.6 (12.4)	28.4 (12.7)	0.141
FEV_1_, % reference	38.3 (14.3)	37 (14.5)	37 (13.8)	0.696
FVC, % reference	64.9 (17.2)	64.1 (17.2)	60.1 (20.8)	0.292
PaO_2_, mmHg	69.2 (18.0)	74.7 (14.0)	79.2 (14.6)	0.008*
PaCO_2_, mmHg	43.2 (8.7)	42.9 (6.5)	43.3 (6.1)	0.944
6MWT				
Meters	345 (105)	354 (79)	352 (83.9)	0.539
SatO_2_ initial, %	92.2 (4.0)	93.1 (3.5)	94.3 (1.9)	0.002*
SatO_2_ end, %	87 (7.8)	87.1 (8.6)	89.7 (5.5)	0.035*

Data are expressed as mean (SD). *p < 0.05 significant (in bold). 6MWT, 6-min walking test; CAT, COPD Assessment Test; Dyspnea mMRC, dyspnea scale modified Medical Research Council; COPD, chronic obstructive pulmonary disease; FEV_1_, forced expiratory volume in one second; FVC, forced vital capacity; LCADL, London Chest Activity of Daily Living scale; PaCO_2_, partial arterial pressure of carbon dioxide; PaO_2_, partial arterial pressure of oxygen.

### 3.3. Inflammatory changes in serum and sputum

Most serum and sputum inflammatory parameters improved after treatment with azithromycin (Table 3). Of note, treatment significantly lowered serum IL-13, IL-6, and TNFR2 levels, but only lowered sputum TNFR2 levels, with no significant correlation found between serum and sputum levels (data not shown). The decreased inflammatory response was more consistent in serial serum determinations.

**Table 3:** Inflammatory changes at baseline and after 3 and 12 months of treatment with azithromycin.

Parameters	SERUM				SPUTUM			
	Baseline	3 mo.	12 mo.	*p*-value	Baseline	3 mo.	12 mo.	*p*-value
Fibrinogen, mmol/L	4.53 (1.4)	3.85 (0.8)	2.91 (0.99)	0.168	–	–	–	–
CRP, mg/L	15.9 (30.2)	9.42 (21)	5.46 (6.08)	0.142	–	–	–	–
Leukocytes, cels/ μL	8,844 (3,128)	7,580 (1,830)	7,000 (1,480)	0.051	–	–	–	–
Eosinophils, cels/μL	188 (167)	200 (152)	223 (167)	0.801	–	–	–	–
TNFR2, pg/mL	1,386 (476)	1,246 (535)	1,148 (419)	0.001*	1,590 (1,978)	1,073 (1,638)	624 (886)	0.005*
IL-13, pg/mL	6.53 (12.5)	5.33 (8.64)	2.21 (2.42)	0.021*	86.1 (83.6)	47.7 (44.1)	69.1 (102)	0.339
IL-5, pg/mL	2.71 (7.69)	2.06 (3.25)	2.24 (1.71)	0.65	58.1 (73.2)	61.8 (91.7)	94.5 (149)	0.124
IL-6, pg/mL	3.8 (4.70)	2.95 (2.81)	1.34 (3.14)	0.003*	1,993 (3,744)	1,653 (1,869)	1,664 (1,969)	0.568
IL-8, pg/mL	9.17 (5.04)	8.88 (3.95)	8.23 (3.26)	0.26	212,852 (648,466)	97,954 (70,014)	98,734 (98,487)	0.169

Data are expressed as mean (SD). *p < 0.05 significant (in bold). CRP, C-reactive protein; IL, interleukin; Mo., months; TNFR2, tumor necrosis factor receptor 2.

### 3.4. Predictors of clinical response in study participants

After analyzing all serum markers and functional variables, an increase in baseline IL-8 predicted a positive response to azithromycin treatment and a reduction in exacerbations (Supplementary Table S1). Among the sputum markers, a high IL-8 level was also independently associated with good clinical response (Supplementary Table S2).

### 3.5. Effect of treatment with azithromycin on COPD exacerbations

**Figure 2:**
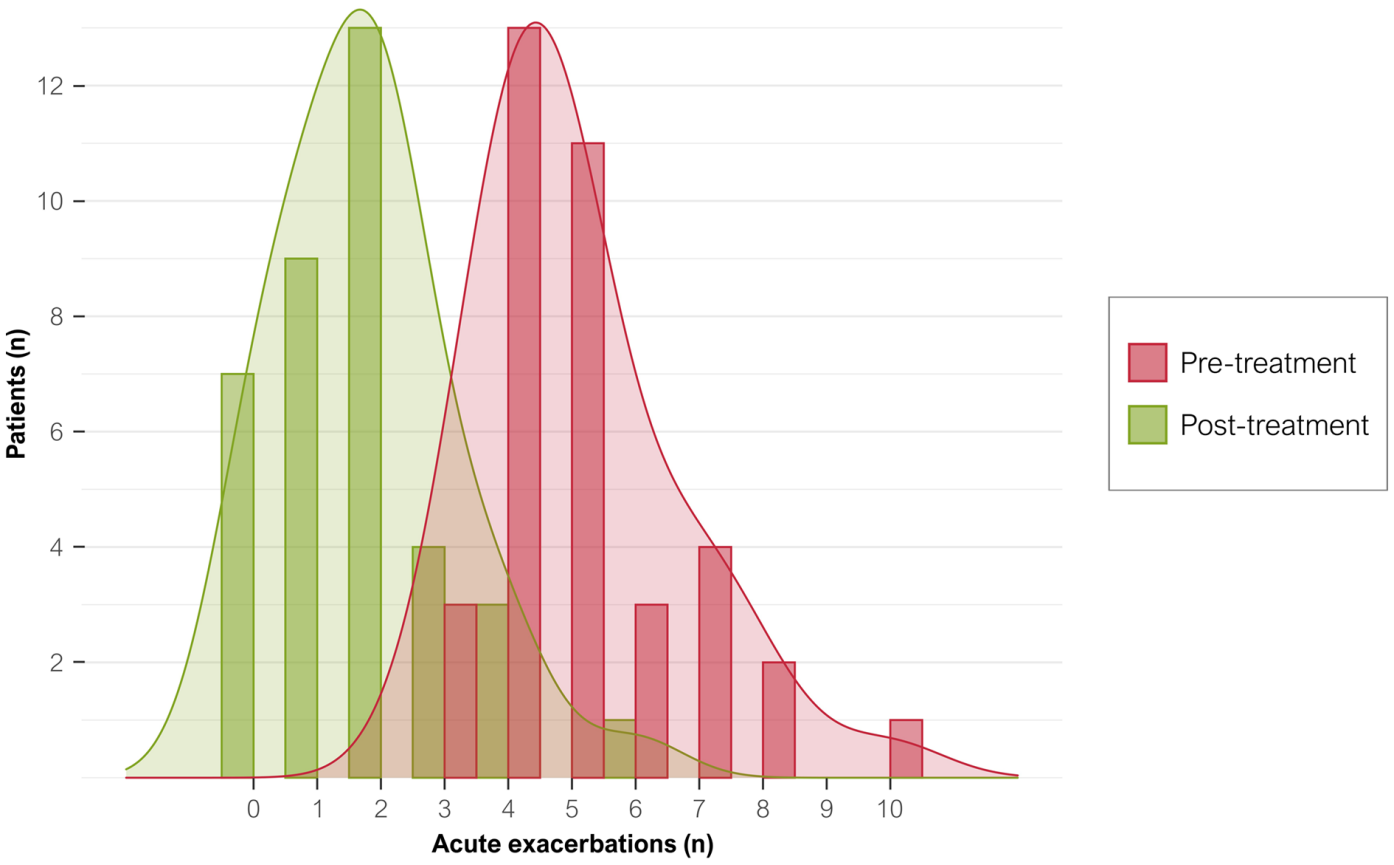
Distribution of exacerbations per patient before and after treatment. Graph shows the number of exacerbations in the year before starting azithromycin treatment (red bars) and after 12 months of treatment (green bars).

The 37 participants had 188 exacerbations (5.1 ± 1.6 AECOPD/patient) in the year before starting azithromycin compared with only 65 during treatment (1.8 ± 1.4 AECOPD/patient; *p* < 0.0001; Figure 2). The IRR of AECOPD after treatment was 0.34 (95%CI, 0.26–0.45), indicating that patients had an approximate 66% reduction in acute exacerbations after treatment. Before treatment, 162 (86%) exacerbations were moderate and 26 (14%) were severe and required hospitalization (mean, 184 days in hospital). During treatment, 55 (84.6%) exacerbations were moderate and 10 (15%) were severe and required hospitalization (mean, 74 days; Table 4). Therefore, hospitalizations fell by 61.5% at 12 months (IRR, 0.39; 95%CI, 0.28–0.49). Although fewer patients required hospital admission, their mean hospital stays tended to be longer (7.7 ± 3.7 vs. 12.3 ± 14.7 days).

**Table 4:** AECOPD characteristics before and after 1 year of azithromycin treatment.

	Pre-treatment	Post-treatment	*p*-value
Patients with AECOPD, *n* (%)	37 (100)	30 (81.1)	0.0054*
AECOPD cases (mean ± SD)	188 (5.1 ± 1.6)	65 (1.8 ± 1.4)	<0.0001*
AECOPD intensity, *n* (%)			
Severe	26 (13.8)	10 (15.4)	0.8370
Moderate	162 (86.2)	55 (84.6)	0.8370
Hospitalization, *n* (%)	26 (13.8)	10 (15.4)	0.8370
Days of hospital stay (mean ± SD)	184 (7.7 ± 3.7)	74 (12.3 ± 14.7)	0.0709
Microbiology pattern AECOPD, *n* (%)			
Cultured samples	91 (48.4)	39 (60.0)	0.1069
Uncultured samples**	97 (51.6)	26 (40.0)	0.1069
Potential pathogenic bacteria (%)^#^			
*Haemophilus influenzae*	28 (30.8)	4 (10.3)	0.0128*
*Moraxella catarrhalis*	22 (24.2)	1 (2.6)	0.0031*
*Pseudomonas aeruginosa*	12 (13.2)	5 (12.8)	0.9547
*Streptococcus pneumoniae*	6 (6.6)	5 (12.8)	0.2424
*Stenotrophomonas maltophilia*	2 (2.2)	4 (10.3)	0.0448*
*Achromobacter xylosoxidans*	2 (2.2)	2 (5.1)	0.3753
*Corynebacterium* spp.	2 (2.2)	2 (5.1)	0.3753
*Enterobacter* spp.	2 (2.2)	0 (0)	0.3508
*Escherichia coli*	2 (2.2)	0 (0)	0.3508
*Serratia marcescens*	2 (2.2)	0 (0)	0.3508
*Citrobacter freundii*	1 (1.1)	0 (0)	0.5111
*Klebsiella oxytoca*	1 (1.1)	0 (0)	0.5111
*Pasteurella multocida*	1 (1.1)	0 (0)	0.5111
*Haemophilus parainfluenzae*	0 (0)	1 (2.6)	0.1252
Normal URT bacterial microbiota	12 (13.2)	17 (43.6)	<0.0001*

**Low quality sputum or no expectoration; #more than one microorganism was isolated in some samples; *p < 0.05 significant (in bold). AECOPD, acute exacerbations of chronic obstructive pulmonary disease; URT, upper respiratory tract.

### 3.6. Microbiological effect of continuous azithromycin treatment

A total of 95 microorganisms were obtained during the 188 AECOPD in the year before starting treatment with azithromycin, which decreased to only 41 microorganisms during 65 AECOPD throughout the follow-up period. There was a significant reduction in frequent sputum isolates, such as *Haemophilus influenzae* (*p* = 0.0128) and *Moraxella catarrhalis* (*p* = 0.0031) (Table 4), and a significant increase in infrequent microorganisms, such as *Stenotrophomonas maltophilia* (from 2.2 to 10.3%; *p* = 0.0448). Microorganisms otherwise increased in the upper respiratory tract, and sputum isolates of *Pseudomonas aeruginosa* and *Streptococcus pneumoniae* did not change.

**Figure 3:**
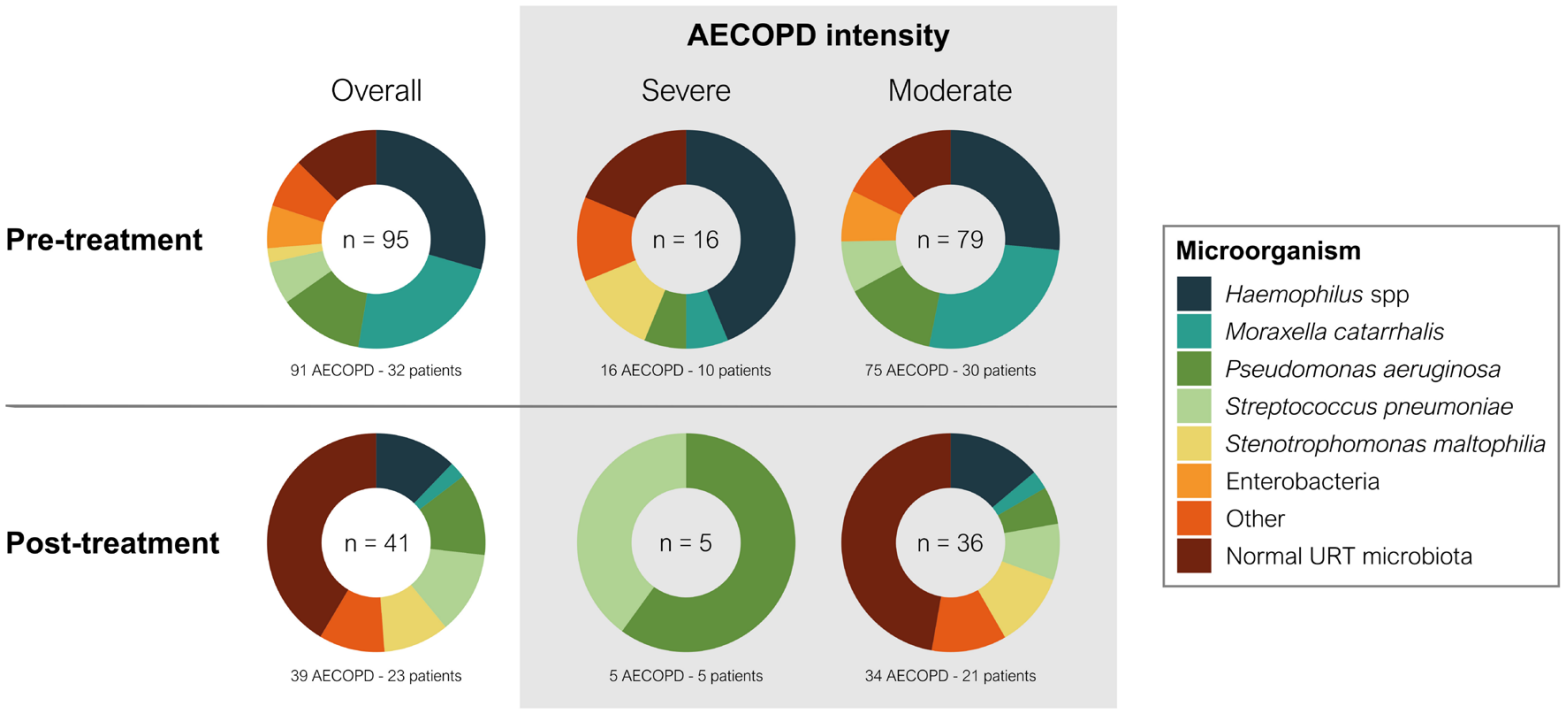
Change of microbiology by AECOPD severity during long-term azithromycin therapy. The number inside each donut chart indicates the number of bacterial isolates. Pre-treatment indicates the year before azithromycin treatment, and post-treatment indicates the first year of azithromycin treatment.

Overall, the pattern of microbiological isolates changed with the intensity of AECOPD from before to after azithromycin therapy (Figure 3). In moderate AECOPD, *M. catarrhalis* decreased from 26.6 to 2.8% (*p* = 0.0026) and *S. maltophilia* increased from 0 to 11.1% (*p* = 0.0026), with a reset observed in the usual upper respiratory tract bacterial microbiota from 11.4 to 47.2% (*p* < 0.0001). In severe AECOPD, *P. aeruginosa* isolates increased from 1 to 3 (*p* = 0.0075) and *S. pneumoniae* isolates increased from 0 to 2 (*p* = 0.0078) after azithromycin therapy.

## 4. Discussion

This multicenter, prospective, observational study analyzed the systemic and functional real-life effects of continuous treatment with azithromycin in patients with severe COPD and frequent exacerbations managed in respiratory day hospitals. In addition to confirming that azithromycin effectively reduced the rate of moderate to severe COPD exacerbations, we provide the first evidence that treatment also improves gas exchange, both at rest and during exercise, after 1 year of treatment. Of the interleukin profiles analyzed in this study, IL-8 and TNFR2 presented as the major inflammatory mediators in the immunomodulatory response to azithromycin in patients with COPD. Baseline elevations of IL-8 in both blood and sputum samples predicted the therapeutic response and may indicate its potential utility as a biomarker. Changes were also observed in the microbial composition of subsequent AECOPD episodes after treatment, with the identification of potentially pathogenic microorganisms with unusual patterns of intrinsic antibiotic resistance that required treatment. This increases our knowledge about how azithromycin treatment affects the inflammatory response and bacteria selection in the airways, together with the implications for future COPD exacerbations.

As expected, continuous azithromycin treatment in patients with severe COPD and frequent exacerbations led to fewer exacerbations (up to 66%) after 1 year of treatment, even using a dosage of 250 mg 3 times a week. From the limited published evidence available (only two clinical trials (5, 6), both carried out in a different profile of COPD patients) does not allow a categorical and unanimous recommendation for the type of patient chosen and the dose to be administered (8). In other chronic inflammatory respiratory diseases, such as bronchiectasis, the dosage of 250 mg 3 times a week for 1 year has been used successfully, minimizing side effects. Moreover, in airways of these patients, this dosage has demonstrated a significant reduction in host neutrophilic inflammatory response (11). The high effectiveness in the control of exacerbations achieved in this study and the few side effects also support the use of this dosage in clinical practice in COPD patients. Previous studies have shown that patients with more severe COPD and higher numbers of exacerbations during the previous year had higher reductions in AECOPD with azithromycin treatment (5–7). These results are consistent with the high effectiveness observed in this study and support the need to identify candidates for preventive therapy accurately and to avoid the use of azithromycin in patients with less severe disease who may not benefit. However, it was notable that neither baseline lung function nor the number of previous exacerbations predicted treatment response in this cohort. Instead, baseline elevations of IL-8 in both blood and sputum samples predicted the therapeutic response, suggesting that this could be a useful biomarker in clinical practice (12, 13).

Another novel finding was the improved arterial oxygenation in patients after 12 months of treatment with azithromycin, both at rest and after exercise, which was associated with effective control of AECOPD. To the best of our knowledge, gas exchange analysis has not been evaluated in studies of azithromycin in patients with COPD. Given that exercise tolerance and lung functional parameters did not improve with azithromycin therapy, this indicates a dissociation between systemic functional data and gas exchange. There are some mechanisms through which azithromycin may improve gas exchange in patients with COPD. The most important is provided by the drug’s anti-inflammatory properties, which reduce the chronic inflammatory response that occurs in the lungs of COPD patients. The decrease in inflammatory markers in blood and sputum after starting prolonged treatment with azithromycin would indicate less airway inflammation and, as a result, an improvement in the pulmonary ventilation-perfusion ratios of treated patients, which presumably contributes to the improvement of gas exchange. In addition, azithromycin can modulate the immune response in the lungs, which may help reduce inflammation; finally, by reducing the frequency and severity of exacerbations, further lung damage can be prevented, leading to more effective lung function.

After analyzing the inflammatory parameters in serum and sputum samples, we observed a numerical improvement in most variables. This was particularly notable in the significant reductions of serum IL-13 and IL-6, as well as serum and sputum TNFR2, after 12 months of treatment. The decreased inflammatory response was more consistent in serial serum determinations than in sputum determinations. Moreover, our predictive models showed that high inflammatory marker and IL-8 levels at baseline predicted a good response to treatment. These immunomodulatory and anti-inflammatory effects of macrolides are well described in the literature (14, 15). Of the interleukin profiles analyzed in this study, IL-8 and TNFR2 presented as the major inflammatory mediators in the immunomodulatory response to azithromycin in patients with COPD. Studies indicate that macrolides take part in proinflammatory cytokine suppression (16–18) and inhibit the transcription and liberation of IL-8, which is important to neutrophil chemotaxis (19). Azithromycin has also been shown to inhibit TNFα-induced production of IL-8 via the JNK signaling pathway and to inhibit MUC5AC production and MMP9. These factors combine to reduce mucus production, which is beneficial for patients with COPD (18). An animal model further showed that TNFα levels were reduced after macrolide treatment, and that this was associated with inhibited neutrophil recruitment in the lungs (12). Other effects of macrolides on cytokines include the modulation of dendritic cells by inhibiting IL-6 and stimulating IL-10 (13). In addition, a potential role of azithromycin in the T helper 2 (Th2) inflammatory pathway has been described, with evidence that it inhibits the expression of different IL- 13-induced genes to reduce mucus expression (i.e., MUC5AC) (20). Overall, our results therefore support previous findings, showing a reduction in cytokine levels associated with neutrophil and eosinophil inflammatory pathways. These help to resolve acute infections and reduce exacerbations in patients with chronic airway diseases.

Despite the significant reduction in exacerbations under continuous azithromycin treatment, some patients still had further AECOPD episodes. Although these patients had fewer hospitalizations, they required more days in hospital to treat severe AECOPD. They also showed changes in the patterns of microbiological isolates in sputum samples after continuous azithromycin treatment, with fewer frequent (*H. influenzae* and *M. catarrhalis*) and more potentially pathogenic microorganisms with unusual patterns of intrinsic antibiotic resistance that required treatment (e.g., *S. maltophilia* or *Achromobacter xylosoxidans* in 15% of isolates). This has clear implications when selecting empirical antibiotics for residual AECOPDs in patients receiving continuous azithromycin. Viral infections usually act as the trigger of the exacerbation, but no routine study of viral infections was performed in our study. Further research is therefore required.

Although it was not the scope of our study, many antibiotic resistance mechanisms have been described among the common microorganisms after continuous macrolide treatment (21–24). Indeed, the antimicrobial properties of azithromycin seem to affect the respiratory microbiota, and this may change the clinical evolution of COPD. Future studies should therefore look at the impact of limiting the use of some antibiotics in future AECOPD episodes based on the resistance mechanisms that develop after continuous azithromycin therapy.

The main limitation of the present study is the small sample size and absence of a control group. However, we did not want to demonstrate the efficacy of the treatment with azithromycin in a clinical trial. Instead, we wanted to analyze the effectiveness of azithromycin in real-world settings among patients with severe COPD (e.g., older, comorbidities, and frequent exacerbations), who clinical trials might miss, and to study the role of azithromycin on inflammation and bacterial isolates in these cases.

In conclusion, continuous azithromycin treatment for patients with severe COPD, comorbidities, and frequent exacerbations not only significantly reduces exacerbation and severity rates after 1 year of treatment but also improves gas exchange. Changes are also seen in microorganism patterns and inflammatory responses after treatment. Of note, high serum and sputum IL-8 levels at baseline also appear to predict a good clinical response to azithromycin and may indicate its potential utility as a biomarker. If proven, IL-8 as could be used to improve patient selection and reduce microbial resistance in clinical practice.

## Data availability statement

The original contributions presented in the study are included in the article/Supplementary material, further inquiries can be directed to the corresponding authors.

## Ethics statement

The studies involving human participants were reviewed and approved by local ethics committee (Comité Ética Investigación Clínica, CEIC, ref. SSP-AZI-2016-01). The patients/participants provided their written informed consent to participate in this study.

## Author contributions

EC participated in patient’s inclusion, statistical analysis, figure preparation, data interpretation, and manuscript drafting. DH participated in the study design, patient’s inclusion, statistical analysis, data interpretation, and obtaining funding. CM and AM participated in the study design, patient’s inclusion, and data interpretation. AC-S participated in sputum analysis, figure and table preparation, and data interpretation. XP participated patient’s inclusion and data interpretation. MG-N participated in sputum analysis and ELISA experiments. SM participated in sputum analysis, data interpretation, and critical revision of the manuscript. SS contributed to the study concept and design, patient inclusion, funding, study supervision, data interpretation, manuscript drafting, and critical revision of the manuscript for important intellectual content. All authors contributed to the article and approved the submitted version.

## Funding

The research was funded by the Instituto de Salud Carlos III (grant PI20/00777 and PI16/00977), co-funded by the European Regional Development Fund (ERDF) “A Way to Build Europe” and grants from Menarini to SS and from SEPAR 088/2016, SEPAR 1116/2020 and FUCAP-2017.

## Conflict of interest

The authors declare that the research was conducted in the absence of any commercial or financial relationships that could be construed as a potential conflict of interest.

## Publisher’s note

All claims expressed in this article are solely those of the authors and do not necessarily represent those of their affiliated organizations, or those of the publisher, the editors and the reviewers. Any product that may be evaluated in this article, or claim that may be made by its manufacturer, is not guaranteed or endorsed by the publisher.

## Abbreviations

6MWT: 6-min walking test
AECOPD: acute exacerbation of COPD
AMI: Acute myocardial infarction
COPD: Chronic obstructive pulmonary disease
CRP: C-reactive protein
ELISA: Enzyme-linked immunosorbent assay
HGTiP: Hospital Germans Trias I Pujol
HPT: Hospital Parc Taulí
HUB: Hospital Universitari de Bellvitge
ICS: inhaled corticosteroids
JNK: c-Jun N-terminal kinase
LABA: long-acting β_2_-agonist
LAMA: long-acting muscarinic antagonist
MMP9: Matrix metalloproteinase 9
MUC5AC: Mucin 5 AC
URT: Upper respiratory tract

## References

[1] Suissa S Dell’Aniello S Ernst P. Long-term natural history of chronic obstructive pulmonary disease: severe exacerbations and mortality. Thorax. (2012) 67:957–63. doi: 10.1136/thoraxjnl-2011-201518, PMID: 22684094 PMC3505864

[2] Celli BR Fabbri LM Aaron SD Agusti A Brook R Criner GJ. An updated definition and severity classification of chronic obstructive pulmonary disease exacerbations: the Rome proposal. Am J Respir Crit Care Med. (2021) 204:1251–8. doi: 10.1164/rccm.202108-1819PP, PMID: 34570991

[3] Marchetti N Criner GJ Albert RK. Preventing acute exacerbations and hospital admissions in COPD. Chest. (2013) 143:1444–54. doi: 10.1378/chest.12-1801 23648908

[4] Ni W Shao X Cai X Wei C Cui J Wang R. Prophylactic use of macrolide antibiotics for the prevention of chronic obstructive pulmonary disease exacerbation: a meta-analysis. PLoS One. (2015) 10:e0121257. doi: 10.1371/journal.pone.0121257, PMID: 25812085 PMC4374882

[5] Albert RK Connett J Bailey WC Casaburi R Cooper JA Jr Criner GJ. Azithromycin for prevention of exacerbations of COPD. N Engl J Med. (2011) 365:689–98. doi: 10.1056/nejmoa1104623, PMID: 21864166 PMC3220999

[6] Uzun S Djamin RS Kluytmans JAJW Mulder PGH van't Veer NE Ermens AAM. Azithromycin maintenance treatment in patients with frequent exacerbations of chronic obstructive pulmonary disease (COLUMBUS): a randomised, double-blind, placebo-controlled trial. Lancet Respir Med. (2014) 2:361–8. doi: 10.1016/S2213-2600(14)70019-0, PMID: 24746000

[7] Pomares X Montón C Huertas D Marín A Cuevas E Casabella A. Efficacy of low-dose versus high-dose continuous cyclic azithromycin therapy for preventing acute exacerbations of COPD. Respiration. (2021) 100:1070–7. doi: 10.1159/000517781 34365450

[8] Global Initiative for Chronic Obstructive Lung Disease I. Global strategy for the diagnosis, management, and prevention of chronic obstructive pulmonary disease. (2020). Available at: (www.goldcopd.org).

[9] Garrod R Bestall JC Paul EA Wedzicha JA Jones PW. Development and validation of a standardized measure of activity of daily living in patients with severe COPD: the London chest activity of daily living scale (LCADL). Respir Med. (2000) 94:589–96. doi: 10.1053/RMED.2000.0786, PMID: 10921765

[10] Domenech A Puig C Martí S Santos S Fernández A Calatayud L. Infectious etiology of acute exacerbations in severe COPD patients. J Infect. (2013) 67:516–23. doi: 10.1016/J.JINF.2013.09.003 24055804

[11] Keir H Shoemark A Dicker A Perea L Pollock J Giam YH. Neutrophil extracellular traps, disease severity, and antibiotic response in bronchiectasis: an international, observational, multicohort study. Lancet Respir Med. (2021) 9:873–84. doi: 10.1016/S2213-2600(20)30504-X, PMID: 33609487

[12] Tsai WC Rodriguez ML Young KS Deng JC Thannickal VJ Tateda K. Azithromycin blocks neutrophil recruitment in pseudomonas endobronchial infection. Am J Respir Crit Care Med. (2004) 170:1331–9. doi: 10.1164/rccm.200402-200OC, PMID: 15361366

[13] Sugiyama K Shirai R Mukae H Ishimoto H Nagata T Sakamoto N. Differing effects of clarithromycin and azithromycin on cytokine production by murine dendritic cells. Clin Exp Immunol. (2007) 147:540–6. doi: 10.1111/j.1365-2249.2007.03299.x, PMID: 17302905 PMC1810497

[14] Friedlander AL Albert RK. Chronic macrolide therapy in inflammatory airways diseases. Chest. (2010) 138:1202–12. doi: 10.1378/chest.10-0196 21051396

[15] Martinez FJ Curtis JL Albert R. Role of macrolide therapy in chronic obstructive pulmonary disease. Int J COPD. (2008) 3:331–50. doi: 10.2147/copd.s681 PMC2629987 18990961

[16] Uli O Erakovi V Parnham MJ. Anti-inflammatory effects of macrolide antibiotics. Eur J Pharmacol. (2001) 429:209–29. doi: 10.1016/S0014-2999(01)01321-8 11698042

[17] Rubin B Tamaoki J. Macrolide antibiotics as biological response modifiers. Curr Opin Investig Drugs. (2000) 1:169–72. PMID: 11249569

[18] Yang J. Mechanism of azithromycin in airway diseases. J Int Med Res. (2020) 48:030006052093210. doi: 10.1177/0300060520932104, PMID: 32589092 PMC7323306

[19] Takizawa H Desaki M Ohtoshi T Kawasaki S Kohyama T Sato M. Erythromycin modulates IL-8 expression in normal and inflamed human bronchial epithelial cells. Am J Respir Crit Care Med. (1997) 156:266–71. doi: 10.1164/ajrccm.156.1.9612065, PMID: 9230759

[20] Mertens TCJ Hiemstra PS Taube C. Azithromycin differentially affects the IL-13-induced expression profile in human bronchial epithelial cells. Pulm Pharmacol Ther. (2016) 39:14–20. doi: 10.1016/J.PUPT.2016.05.005, PMID: 27246785

[21] Roberts MC. Update on macrolide-lincosamide-streptogramin, ketolide, and oxazolidinone resistance genes. FEMS Microbiol Lett. (2008) 282:147–59. doi: 10.1111/J.1574-6968.2008.01145.X 18399991

[22] Serisier DJ. Risks of population antimicrobial resistance associated with chronic macrolide use for inflammatory airway diseases. Lancet Respir Med. (2013) 1:262–74. doi: 10.1016/S2213-2600(13)70038-9, PMID: 24429132

[23] Yamaya M Azuma A Takizawa H Kadota JI Tamaoki J Kudoh S. Macrolide effects on the prevention of COPD exacerbations. Eur Respir J. (2012) 40:485–94. doi: 10.1183/09031936.00208011 22408201

[24] Carrera-Salinas A González-Díaz A Ehrlich RL Berbel D Tubau F Pomares X. Genetic adaptation and acquisition of macrolide resistance in haemophilus spp. during persistent respiratory tract colonization in chronic obstructive pulmonary disease (COPD) patients receiving long-term azithromycin treatment. Microbiol Spectr. (2023) 11:e0386022. doi: 10.1128/SPECTRUM.03860-22, PMID: 36475849 PMC9927455

