# Supplementary material for: Systemic and functional effects of continuous azithromycin treatment in patients with severe chronic obstructive pulmonary disease and frequent exacerbations
Source: Front Med (Lausanne). 2023 Jul 24;10:1229463. doi: 10.3389/fmed.2023.1229463 (PMC10406447; doi:10.3389/fmed.2023.1229463)
Supplement: Supplementary file 1 [file Table_1.DOCX]

**SUPPLEMENTARY MATERIAL**

**Supplementary Table S1. Effect of baseline clinical, functional, and serum inflammatory variables on therapeutic response.**

|  | **IRR** | **95%CI** | **p-value** |
| --- | --- | --- | --- |
| **Fibrinogen** | 1.14 | 0.96–1.31 | 0.092 |
| **CRP** | 0.94 | 0.73–1.13 | 0.581 |
| **Leukocytes** | 1.05 | 0.46–2.19 | 0.899 |
| **IL-13** | 1.03 | 0.84–1.24 | 0.747 |
| **IL-5** | 1.02 | 0.82–1.23 | 0.834 |
| **IL-6** | 0.98 | 0.76–1.2 | 0.849 |
| **IL-8** | 1.27 | 1.08–1.46 | **0.001*** |
| **TNFR2** | 0.98 | 0.79–1.21 | 0.88 |
| **6MWT, meters** | 0.97 | 0.8–1.18 | 0.763 |
| **PaO_2_** | 1.14 | 0.94–1.39 | 0.19 |
| **FVC%** | 1.08 | 0.8–1.46 | 0.611 |
| **FEV_1_%** | 1.36 | 0.94–1.97 | 0.093 |
| **Previous AECOPD** | 1.36 | 0.94–1.97 | 0.093 |

Incidence Rate Ratio (IRR) adjusted for age and Charlson; 95% confidence interval (95%CI). *P<0.05 significant.

**Supplementary Table S2. Effect of baseline sputum interleukin levels on therapeutic response (in number of exacerbations).**

|  | **IRR** | **95%CI** | **p-value** |
| --- | --- | --- | --- |
| **IL-1b** | 0.82 | 0.58–1.08 | 0.189 |
| **IL-6** | 0.98 | 0.76–1.2 | 0.849 |
| **IL-8** | 1.27 | 1.08–1.46 | **0.001*** |
| **IL-13** | 1.03 | 0.84–1.24 | 0.747 |
| **TNFR2** | 0.98 | 0.79–1.21 | 0.88 |

Incidence Rate Ratio (IRR) adjusted for age and Charlson; 95% confidence interval (95%CI). *P<0.05 significant.
